# Role of the gut microbiota in the pathogenesis of endometriosis: a review

**DOI:** 10.3389/fmicb.2024.1363455

**Published:** 2024-03-05

**Authors:** Cuishan Guo, Chiyuan Zhang

**Affiliations:** Department of Obstetrics and Gynecology, Shengjing Hospital of China Medical University, Shenyang, China

**Keywords:** gut microbiota, endometriosis, estrogen, inflammation, immunity

## Abstract

Endometriosis is classically defined as a chronic inflammatory heterogeneous disorder occurring in any part of the body, characterized by estrogen-driven periodic bleeding, proliferation, and fibrosis of ectopic endometrial glands and stroma outside the uterus. Endometriosis can take overwhelmingly serious damage to the structure and function of multi-organ, even impair whole-body systems, resulting in severe dysmenorrhea, chronic pelvic pain, infertility, fatigue and depression in 5–10% women of reproductive age. Precisely because of a huge deficiency of cognition about underlying etiology and complex pathogenesis of the debilitating disease, early diagnosis and treatment modalities with relatively minor side effects become bottlenecks in endometriosis. Thus, endometriosis warrants deeper exploration and expanded investigation in pathogenesis. The gut microbiota plays a significant role in chronic diseases in humans by acting as an important participant and regulator in the metabolism and immunity of the body. Increasingly, studies have shown that the gut microbiota is closely related to inflammation, estrogen metabolism, and immunity resulting in the development and progression of endometriosis. In this review, we discuss the diverse mechanisms of endometriosis closely related to the gut microbiota in order to provide new approaches for deeper exploration and expanded investigation for endometriosis on prevention, early diagnosis and treatment.

## 1 Introduction

Endometriosis is characterized as a common estrogen-dependent chronic inflammatory gynecological disorder that affects 6–10% women during their reproductive period (Saunders and Horne, 2021). It is defined that endometrial glands and stroma growing outside the uterus undergo periodic bleeding, proliferation, and fibrosis due to estrogen, ultimately influencing women’s health, quality of life, work productivity and creating serious economic burden (Fuldeore et al., 2011). Endometriosis might be better considered a “syndrome,” either because it is closely related to a wide range of symptoms, including severe dysmenorrhea, chronic pelvic pain, heavy menstruation, bowel and bladder symptoms, fatigue and depression, or because very little is known about endometriosis due to its high symptom heterogeneity and increasingly apparent complexity of pathogenesis (Saunders and Horne, 2021). In addition, there may be delays of up to 4–11 years for a definitive diagnosis of endometriosis because of atypical symptoms and the lack of specific diagnostic markers (Taylor et al., 2021). Treatment options for endometriosis include surgical resection of lesions and medical therapy of ovarian suppression. High recurrence rate after surgery and side-effects attributed to drugs make treatment of disease a tricky challenge. Thus, further exploration is required to understand the underlying pathogenesis of endometriosis, involving immunity, inflammation, metabolomics, endocrinology and changes of pelvic environment (Olovsson, 2011), and design new treatment strategies.

Increasing studies have indicated that the gut microbiota plays a significant role in various critical biological processes and the development of human diseases, involving in the body’s immunity, metabolism, inflammation, hormone regulation, and even brain regulation (Wang et al., 2023), which are closely related to the occurrence and development of inflammatory bowel disease (IBD) (Shan et al., 2022), diabetes (Yang et al., 2021), polycystic ovary syndrome (PCOS) (Liang et al., 2021), ovarian cancer (Borella et al., 2021) and depression (Simpson et al., 2021; Hong et al., 2023). One study of monkeys found that monkeys with endometriosis had a different profile of shed microflora (lower *Lactobacilli* and higher *Gram-negative bacteria*) and the incidence of intestinal inflammation was higher than that in healthy controls (Bailey and Coe, 2002). Furthermore, 16S rRNA gene sequencing technology was used to compare the fecal bacteria between mice with and without endometriosis, and the result showed mice with endometriosis were different from the control group at various classification levels, showing a general decrease in the level of *Bacteroides* (Yuan et al., 2018). Another study reported the growth of the ectopic endometriotic lesion were reduced in microbiota-depleted (MD) mice compared to control mice, and could be restored through oral gavage with feces from mice with endometriosis (Chadchan et al., 2023). Although the exact mechanisms are unknown, it is possible that there is a certain correlation between endometriosis and gut microbiota. In addition, intestinal microorganisms have also been proved to be involved in the regulation of estrogen (Baker et al., 2017). Immune dysregulation, inflammatory response and abnormal estrogen metabolism caused by the imbalance of intestinal homeostasis may stimulate the growth of endometriosis and promote the development and progression of endometriosis (Hantschel et al., 2019).

In this review, we discuss a wide variety of mechanisms of endometriosis that the gut microbiota may be involved in, aiming to better understand the complex pathogenesis of endometriosis and provide microbiome-based approaches for prevention, early diagnosis and treatment of endometriosis for the future.

## 2 Gut microbiota

In recent years, growing evidence suggests that gut microbiota plays a crucial role in various diseases. The gut flora of adult weighs more than 1 kg, and the number of bacteria is as high as 100 trillion (10^14^), encoding about 5 million genes, more than 150 times that of the human genome-encoding gene (D'argenio and Salvatore, 2015). The distribution of intestinal microbes is characterized by diversity and complexity. The number and types of bacteria from the stomach to the colon are gradually increasing, and the dominant species distributed in each part are also different with each other. The large intestine is the main site of permanent microbial colonization in the human body. With the growth of the human body, the intestinal flora in the body continues changing, but eventually stabilizes (Claesson et al., 2011). It is reported that *Firmicutes* and *Bacteroidetes* were the most important phyla of intestinal flora, followed by *Proteobacteria*, *Actinobacteria*, *Fusobacteria*, and *Verrucomicrobia* (Eckburg et al., 2005). The gut microbiota can not only synthesize a variety of vitamins (such as vitaminB1, B2, B6, and B12, and vitamin K), folic acid and amino acids necessary for body growth, but also promote the absorption of mineral elements and help digestion of metabolic foods such as complex polysaccharides, fats, bile acids, and so on (Liu et al., 2021; Du et al., 2022). Besides, it can promote angiogenesis and epithelial repair (Ramakrishna, 2013; Schirbel et al., 2013; Abdollahi-Roodsaz et al., 2016), boost maturation of the immune system (Al-Rashidi, 2022), influence brain-gut communication (Mayer et al., 2022), and protect the host from pathogen infection. Gut microbial genetic variation also modulates host lifespan, sleep, and motor performance (Li et al., 2023).

Studies have shown that the composition of the intestinal flora can reflect certain behavioral characteristics of the host to some extent, such as diet, environmental exposure, antibiotic use (Fourie et al., 2017). Healthy human intestinal microbes, the external environment and immune status of the host are in a dynamic balance, which helps maintaining the body’s normal immune defense function. When the balance is broken, there will be gut dysbiosis, usually manifested by a decrease diversity of bacterial species and the presence of pathobionts, which will lead to various diseases ranging from gastrointestinal and metabolic conditions to immunological and neuropsychiatric diseases (Ratajczak et al., 2019).

## 3 The gut microbiota of endometriosis

Previous researchers have shown that animal models of endometriosis and patients with endometriosis have gut dysbiosis (Table 1). However, some reported results are not completely consistent (Iavarone et al., 2023; Ser et al., 2023). Female C57BL/6 J mice were commonly used to construct the endometriosis mouse model. One study reported the abundance of *Firmicutes* increased and *Bacteroid Ota* decreased through establishing an endometriosis mouse model by intraperitoneal injection of allogeneic mouse endometrium and 21 days of endometriosis induction (Ni et al., 2021). Another study came to the opposite conclusion, with decreased abundance of *Firmicutes* and increased abundance of *Bacteroidetes* (Chadchan et al., 2019). In addition, still another study showed that they did not find that the compositions of gut microbiota were abnormal until 42 days after modeling, using the same method to build a mouse endometriosis model. The result *Firmicutes* were enriched in the endometriosis group, whereas *Bacteroidetes* were enriched in the control group. The ratio of *Firmicutes*/*Bacteroides* was about twice as high as the control (Yuan et al., 2018). Moreover, Hantschel et al. found that gut microbiota composition didn’t appear to be affected by endometriosis induction at 21 days after the modeling, suggesting no signs of gut dysbiosis during the early stages of lesion formation, which is consistent with Yuan’s results (Hantschel et al., 2019). The above-mentioned animal experimental results are inconsistent, which may be related to differences in animal sources, modeling methods, feeding conditions, and sampling time.

**Table 1:** Features of gut microbiota changes in animals and humans with endometriosis in previous studies.

Type	Detection technique	Results	References
Rhesus monkey	Differential and selective agars method for enumeration of gut microflora	Lactobacilli ↓ Gram-negative bacteria ↑	Bailey and Coe (2002)
Mice	Bacterial 16S rRNA gene sequencing	Bacteroidetes ↑ Firmicutes ↓	Chadchan et al. (2019)
Mice	V4 regions of the 16S rRNA gene	Firmicutes/ Bacteroidetes ↑ At the phylum level: *Firmicutes* and *Actinobacteria* ↑ At the class level: *unidentified Actinobacteria* and *Betaproteobacteria* ↑ At the order level: *Bifidobacteriales* and *Burkholderiales* ↑ At the family level: *Bifidobacteriaceae and Alcaligenceae* At the genus level: *Ruminococcaceae*-UGG-014, *Bifidobacterium* and *Parasutterella* ↑	Yuan et al. (2018)
Mice	V4 and V5 region of bacterial 16S rRNA genes	No significant difference	Hantschel et al. (2019)
Mice	16S rRNA sequencing	At the phylum level: Firmicutes ↑, Bacteroidetes ↓ Actinobacteriota and Patescibacteria ↑ Deferribacterota, Campilobacterota and Desulfobacterota ↓ At the genus level: *Lactobacillus, Clostridium_sensu_stricto_1, Bifidobacterium* and *Candidatus_Saccharimonas* ↑ *Bacteroides, Dubosiella* and *Muribaculum* ↓	Ni et al. (2021)
Mice	16S rRNA sequencing	At the phylum level: *Firmicutes* ↓, *Bacteroides* ↓ *Firmicutes*/*Bacteroides* ↓ *Proteobacteria* ↑, *Verrucomicrobia* ↑ At the genus level: *Allobaculum*, *Akkermansia*, *Parasutterella,* and *Rikenella* ↑ *Lachnospiraceae_NK4A136_* *group*, *Lactobacillus*, *Bacteroides* ↓	Ni et al. (2020)
Mice	V3 and V4 regions of the 16 s rDNA sequences were amplified	At the phylum level: *Firmicutes* ↑ *Bacteroidetes* and *Proteobacteria* ↓ At the class level: *Bacilli* ↑ *Clostridia* and *Bacteroidia* ↓ At the family level: Lactobacillaceae ↑ *Ruminococcaceae* and *Peptostreptococcaceae* ↓ At the genus level: *Lactobacillus* ↑	Cao et al. (2020)
Female olive baboons	V4 region of the 16S rRNA gene	Firmicute/Bacteroidetes ↑ The levels of *Succinivibrio, Prevotella, Megasphaera, Lactobaccillus* and CF231 decreased at 3 months post-inoculation, but the levels of *Succinivibrio, Prevotella*, and CF231 increased throughout disease progression from 6 to 9 months post-inoculation.	Le et al. (2022)
Human	The V1–V3 regions of the 16S ribosomal RNA gene	α and β diversities ↓ At the genus level: *Bacteroides, Parabacteroides, Oscillospira* and *Coprococccus* ↑ *Paraprevotella, Lachnospira and Turicibacter* ↓	Svensson et al. (2021)
Human	The V3 and V4 regions of the 16S rRNA gene were amplified	At the genus level: *Sneathia, Barnesella* and *Gardnerella* ↓	Ata et al. (2019)
Human	V3-V4 variable region of the bacterial 16sRNA	α diversity ↓ At the phylum level: *Firmicutes/Bacteroidetes ↑* *Actinobacteria, Cyanobacteria, Saccharibacteria, Fusobacteria,* and *Acidobacteria* ↑ *Tenericutes* ↓ At the genera level: *Bifidobacterium, Blautia, Dorea, Streptococcus, and [Eubacterium] hallii_group* ↑ *Lachnospira* and *[Eubacterium] eligens_group* ↓	Shan et al. (2021)
Human	V4 regions of the 16S rRNA gene were amplified and sequenced	Clostridia Clostridiales, Lachnospiraceae Ruminococcus, Clostridiales Lachnospiraceae, Ruminococcaceae Ruminococcus, Lachnospiraceae Dorea ↓ *Eggerthella lenta and Eubacterium dolichum* ↑	Huang et al. (2021)

Both α and β diversities of the gut microbiota were significantly reduced in patients with endometriosis. A higher abundance of *Bacteroides*, *Parabacteroides*, *Oscillospira* and *Coprococccus*, and a lower abundance of *Paraprevotella*, *Lachnospira*, and *Turicibacter* were detected (Svensson et al., 2021). Furthermore, in patients with stage III/IV endometriosis, *Sneathia*, *Barnesella* and *Gardnerella* were significantly decreased at the genus level (Ata et al., 2019). Another study showed that the ratio of *Firmicutes*/*Bacteroidetes* was increased and *Blautia*, *Bifidobacterium*, *Dorea*, and *Streptococcus* were significantly increased in patients with stage III/IV endometriosis (Shan et al., 2021). Huang et al. also found that *Clostridia Clostridiales, Lachnospiraceae Ruminococcus*, *Clostridiales Lachnospiraceae*, *Ruminococcaceae Ruminococcus*, and *Lachnospiraceae Dorea* were significantly reduced in patients with endometriosis (Huang et al., 2021).

Although all of these studies have focused on finding distinctive features of gut microbiota changes in patients with endometriosis, there is a lack of consensus among their results, possibly related to inconsistencies in diagnostic criteria, grouping criteria, and methods of fecal microflora detection. And the gut is a complex ecosystem, which may be affected by various factors, such as genetics, diet, age, regional differences, and many more. However, the results presented above all indicate that the gut microbiota is associated with endometriosis. Further large-scale clinical studies are needed to clarify their clinical significance.

## 4 The possible mechanism of gut microbiota involved in the endometriosis

**Figure 1:**
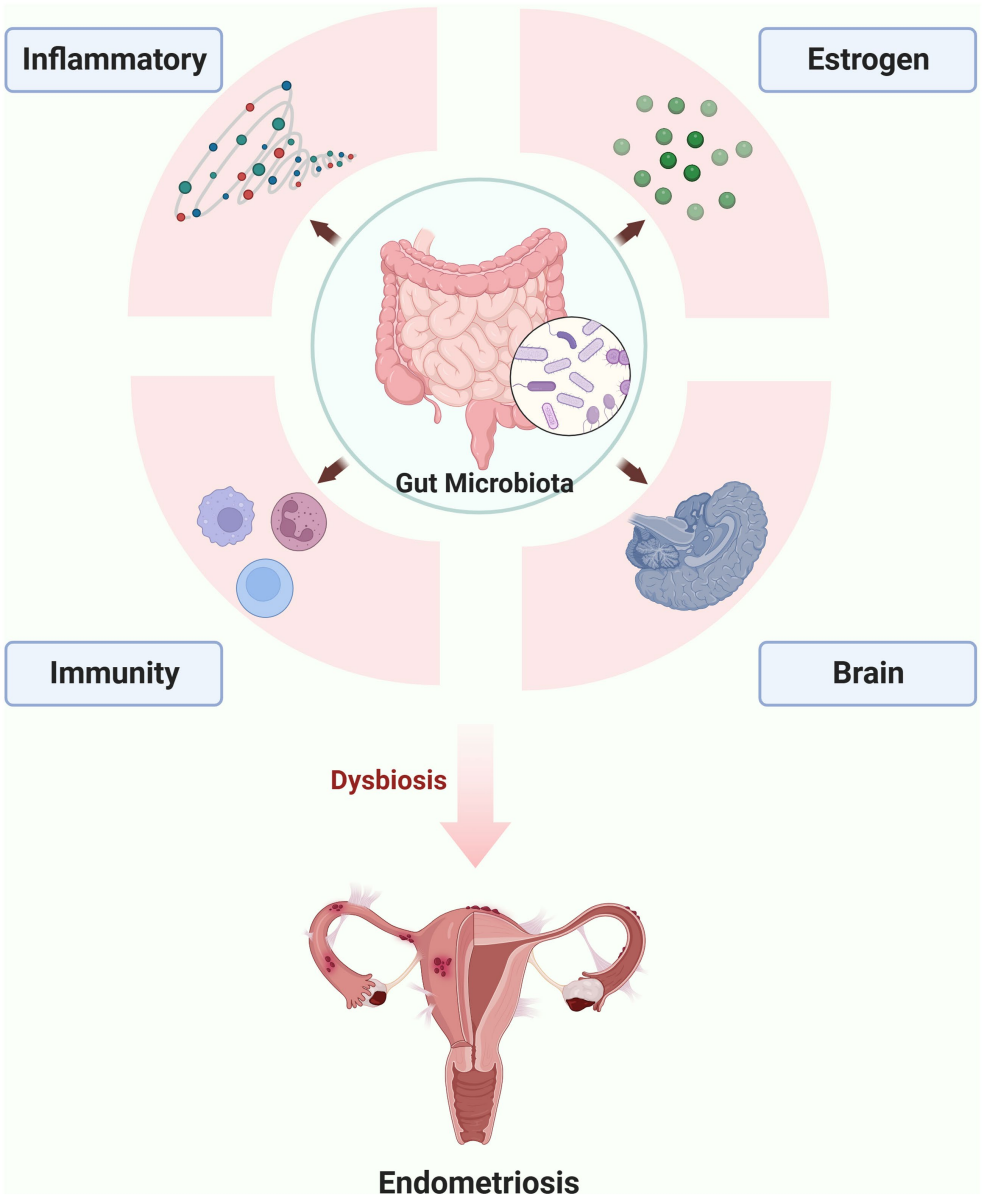
The possible mechanism of gut microbiota involved in the endometriosis (the figures in this review were created with BioRender.com).

To elucidate the underlying mechanism of gut microbiota involved in the endometriosis, the following aspects of immunity, inflammatory, Estrogen-Gut microbiome axis in endometriosis and Gut-Brain Axis in endometriosis are discussed in greater detail in the following sections (Figure 1).

### 4.1 Immunity

The intestine is one of the organs with the most immune cells in the human body. The gastrointestinal-associated lymphoid tissue can manage the immune system to respond to large amounts of antigen exposure in the intestine and activate adaptive immune responses. The gut microbiota has a strong immune regulation ability, which can induce a systemic immune response, maintain the stability of the intestinal microecology, inhibit the reproduction of pathogenic bacteria, and decompose harmful substances to maintain homeostasis of the host. A large number of microorganisms exist in the intestinal mucosa, which are the source of pathogen-associated molecular patterns (PAMPs) and metabolites. PAMPs including lipopolysaccharide (LPS), peptidoglycans, flagellin and lipoprotein and metabolites including short-chain fatty acids (SCFAs) and bile acids and others were described to play an important role in this aspect (Correa-Oliveira et al., 2016). PAMPs mediate interactions between the immune system and the commensal microflora by binding to pattern recognition receptors (PRRs) [such as Toll-like receptors (TLRs) which are widely expressed on intestinal epithelial cells (IECS) and immune cells (Belizario and Faintuch, 2018)]. The interaction between them can promote the secretion a series of proinflammatory chemokines (IL-8), cytokines (IL-1, IL-6, IL-7, IL-11, and TNF), and growth factors (SCF and G-CSF). These molecules will recruit peripheral neutrophils and mast cells to the subcutaneous area of the intestine, and precipitate activation and differentiation of local lymphocytes (Zhang C. X. et al., 2019). However, the molecular mechanisms to explain microbiota-immune system interactions are not entirely clear.

Gut dysbiosis and bacterial metabolites can lead to disruption of the intestinal barrier, translocation of bacteria and endotoxins, dysregulation of the immune system, resulting in oxidative stress and inflammatory responses, thereby increasing the prevalence of various autoimmune diseases. Many researchers have worked to link autoimmune diseases to endometriosis because endometriosis shares features with some autoimmune diseases, including polyclonal B cell activation, T cells, and B cells dysfunction, decreased apoptosis, tissue damage, and multiorgan involvement, and endometriosis is often associated with autoimmune diseases (Shigesi et al., 2019).

Gut microbiota can influence the differentiation of T lymphocytes into different types of helper T lymphocytes (Th1, Th2, and Th17) or regulatory T lymphocytes (Treg cells), such as *segmented filamentous bacteria* (*SFB*) directly stimulating Th17 differentiation (Ivanov et al., 2009; Wang et al., 2019), *Clostridium* spp. participating in inducing Treg production (Atarashi et al., 2011), and *Bacteroides* involving in regulating Th1/Th2 balance (Mazmanian et al., 2005).

Many studies have shown that Th17 cells and their cytokine profiles are significantly increased in peritoneal fluid of women with endometriosis, and excessive IL-17 from Th17 cells is related to the severity of the disease (Gogacz et al., 2016; Tarokh et al., 2019). Shan J et al. found that compared with the healthy group, the people with stage III/IV endometriosis had a lower α diversity of gut microbiota and a higher Firmicutes/Bacteroidetes ratio which is widely accepted as a feature of dysbiosis. They also found that IL-17A was significantly decreased in the patients with endometriosis which was negatively correlated with the abundances of *Streptococcus* and *Bifidobacterium* (Shan et al., 2021). Le et al. detected that microbiome diversity and abundance were altered at each baboon induced of endometriosis. The induction of endometriosis decreased peripheral Tregs cells while Th17 cells increased at all post-induction collections that caused an immune shift toward an inflammatory profile and altered mucosal microbial profiles (Le et al., 2022). More and more evidences suggest that the abnormal quantity and activity of Treg cells can lead to dysregulation of the immune microenvironment in the body, decreased pelvic immune function, and immune escape, thereby promoting the implantation and growth of ectopic endometrium outside the uterus. Chadchan et al. also observed that there are fewer total and CD206+ (M2-like macrophage) macrophages in the peritoneum of microbiota-depleted (MD) mice compared to vehicle-treated mice. They also found the number of CD19+ B cells, Total T cells, CD4+ T cells, and CD8+ T cells were lower in MD mice than in the vehicle-treated mice. These results suggest that the gut microbiota promotes progression of endometriosis by influencing peritoneal immune cell populations (Chadchan et al., 2023). We can speculate that the onset and development of endometriosis may be related to the abnormal immune response caused by gut dysbiosis.

#### 4.1.1 LPS

LPS is an important part of the outer membrane of Gram-negative bacteria with highly immunogenic and can cause a strong immune response of host. When the gut microbiota are imbalanced, Gram-negative bacteria proliferate in large numbers, and the permeability of the intestinal wall is increased simultaneously. The leakage of bacterial endotoxin LPS is an important indication of gut dysbiosis and intestinal barrier damage (Saad et al., 2016). One Study revealed that the fecal microbiota transplantation from endometriosis mice could destroy the mouse’s intestinal wall barrier and significantly increase the peritoneal LPS level (Ni et al., 2021). Meanwhile, another study observed that the abundance of *Pseudomonadaceae Pseudomonas* and *Prevotellaceae Prevotella*, which could release LPS, increased in the peritoneal cavity of patients with endometriosis (Raia et al., 2005; Huang et al., 2021).

**Figure 2:**
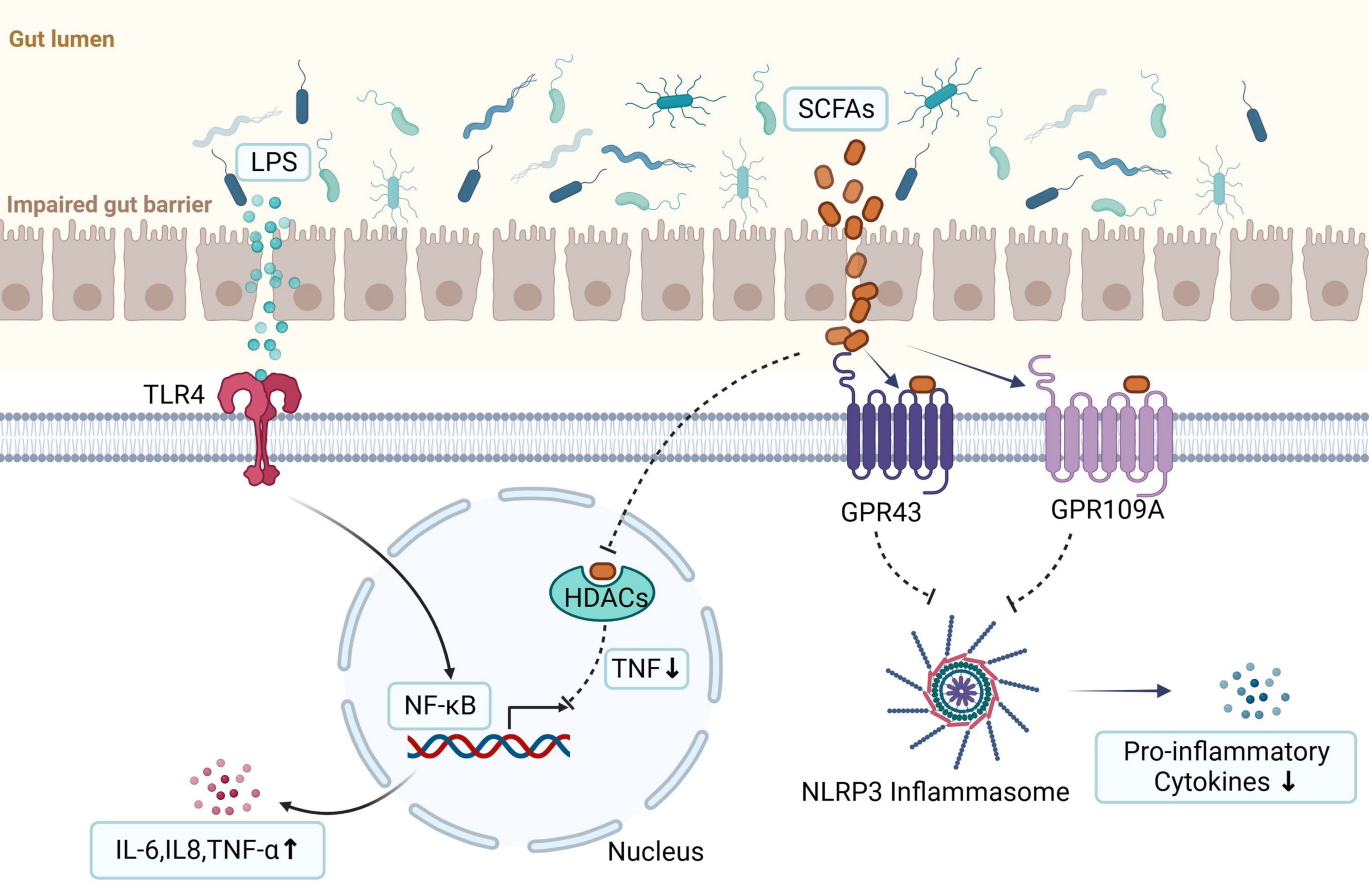
LPS can promote the occurrence and progression of endometriosis via binding with Toll-like receptor 4 (TLR4) and then activate NF-κB. The activated NF-κB enters the nucleus, triggering the transcription of cell adhesion, proliferation, and anti-apoptotic related genes, such as TNF-α, IL-8, IL-6, et al. They are produced by activation of innate immunity and are important contributors to the development of endometriosis. Short chain fatty acids (SCFAs) can exert anti-inflammatory effects through two signal pathways. First, they activate the G protein-coupled receptors (GPCRs) (such as GPR41, GPR43, and GPR109a) and then inhibit the activation of the Nod-like receptor pyrin domain 3 (NLRP3) inflammasome, thereby reducing the secretion of pro-inflammatory cytokines. Second, they can inhibit histone deacetylases (HDACs) to reduce production of the proinflammatory tumor necrosis factor (TNF) which can lead to inactivation of nuclear factor kappa B (NF-κB) (the figures in this review were created with BioRender.com).

LPS can work through the classic toll-like receptor signaling pathway, which has been shown to promote the occurrence and progression of endometriosis via binding with Toll-like receptor 4 (TLR4) (Khan et al., 2010) (Figure 2). TLR4 is a pattern recognition receptor that regulates immune response and inflammatory response in cells, and is widely expressed on the cell membrane of immune cells (such as macrophages, dendritic cells, neutrophils, and natural killer cells). When LPS was released into the bloodstream or other body fluids, it was captured by LPS-binding protein immediately and delivered to TLR4, and then activated NF-κB through both MyD88-dependent and MyD88-independent pathways. The activated NF-κB entered the nucleus, causing immune and inflammatory responses, and triggering the transcription of cell adhesion, proliferation, and anti-apoptotic related genes, such as TNF-α, IL-8, IL-6, and so on.

In mammals, innate immune cells (such as macrophages, dendritic cells) can be activated by bacterial endotoxin or LPS. Previous study demonstrated that bacterial endotoxin LPS could be a potential inflammatory mediator of macrophages (Mφ) stimulation and product different cytokines and growth factors such as hepatocyte growth factor (HGF), vascular endothelial cell growth factor (VEGF), IL-6, TNF-α in the pelvic environment (Khan et al., 2010). They also found that combined treatment with 17β-estradiol and LPS promoted IL-6 and TNF-α secretion by macrophages in the peritoneal fluid of patients with endometriosis, and the growth of eutopic and ectopic endometrial cells. When the effect of estrogen receptor (ER) and TLR4 are blocked, the secretion of inflammatory cytokines and the growth of endometrial cells are significantly inhibited. This is suggestive of an additive effect of estrogen and LPS on promoting pro-inflammatory response in the pelvis and growth of endometriosis (Khan et al., 2015). Moreover, another study has reported that LPS could stimulate endometriotic stromal cells (ESCs) which were obtained from chocolate cyst linings of the ovary to produce significant amounts of TNF-α and IL-8, and promote ESCs proliferation. Anti-TNF-alpha antibody and anti-IL-8 antibody inhibited the stimulatory effects of LPS. NF-κB inhibitor significantly decreased LPS-induced IL-8 protein production and LPS-induced ESCs proliferation (Iba et al., 2004). This indicates that as an initial inflammatory mediator, LPS could be involved in pelvic inflammation and promote the TLR4/ NF-κB mediated growth of endometriosis. Therefore, it is speculated that the gut microbiota disorder can affect the occurrence and development of endometriosis by impacting upon the host’s local innate immune status.

#### 4.1.2 Short chain fatty acids

SCFAs are by-products of gut flora fermentation of dietary fibers, including acetate, propionate, and butyrate and so on. In addition to providing energy for the host and gut bacteria, SCFAs have a variety of other functions, including resisting the invasion of intestinal pathogens, maintaining the intestinal epithelial barrier, regulating host metabolism and immune responses, and enhancing anti-inflammatory and anti-proliferative and even tumor protection (Zhang Z. et al., 2019; Kimura et al., 2020; Borella et al., 2021; Chadchan et al., 2021). The intestinal epithelial barrier populated by innate and adaptive immune cells isolates the inside of the body from the external environment and maintains microorganisms homeostatic to prevent inflammation. However, if this barrier is broken, mechanisms of innate and adaptive immune responses trigger microorganism and tissue repair. SCFAs are regarded as mediators in the communication between the intestinal microbiome and the immune system which act not only locally in the intestines colonized by commensal bacteria, but also affect the intestinal immune cells, and modulate immune response of distant tissues (Fellows et al., 2018).

The levels of SCFAs in the intestines are substantially influenced by the composition of gut microbiota. SCFAs work mainly through two signal pathways. First, they downregulate downstream inflammatory responses by activating G protein coupled receptors (GPCRs) (such as GPR41, GPR43, and GPR109a) which are present in a variety of gut enteroendocrine cells and immune cells. Second, they can inhibit histone deacetylases (HDACs) which are involved in regulation of expression of genes. Recent data found SCFAs could act on mononuclear blood cells and neutrophils by inhibition of HDACs and reduce production of the proinflammatory tumor necrosis factor (TNF) which can lead to inactivation of nuclear factor kappa B (NF-κB) (Correa-Oliveira et al., 2016; Rooks and Garrett, 2016) (Figure 2).

A recent study found that *Lachnospiraceae Ruminococcus* was significantly depleted in feces of patients with endometriosis which positively correlated with SCAFs, especially the biosynthesis of butyrate (Jin et al., 2019; Huang et al., 2021). Additionally, another study reported that altered gut microbiota promoted endometriotic lesion growth and feces from mice with endometriosis contained less of SCFAs and n-butyrate than feces from mice without endometriosis. N-butyrate inhibited human endometriotic cell and lesion growth through GPR43 and GPR109A (Chadchan et al., 2021). We consider that the reduction of *Lachnospiraceae Ruminococcus* may lead to a decrease in the concentration of SCFAs in the human intestine, resulting in the occurrence of diseases.

Moreover, a murine model study showed that butyrate can prevent vascular inflammation or atherosclerosis by inhibiting the Nod-like receptor pyrin domain 3 (NLRP3) inflammasome formation and activation (Yuan et al., 2018). The NLRP3 inflammasome is composed of the sensor protein, NLRP3, the adaptor protein apoptosis-associated speck-like protein (ASC), and the pro-inflammatory caspase-1, which promotes the secretion of pro-inflammatory cytokines, such as bioactive interleukin-1β (IL-1β) and interleukin-18 (IL-18) (Strowig et al., 2012). Meanwhile, Han et al. found that NLRP3 is activated in patients with endometriosis and enhances IL-1β signaling, which can promote the adhesion and proliferation of endometrial cells, and eventually facilitates the formation of ectopic lesions. They also found that ectopic lesion volume was greatly reduced in NLRP3-deficient mice compared with controls (Han et al., 2015; Leavy, 2015). Thus, we revealed that butyrate may have beneficial effects against endometriosis by inhibiting the activation of NLRP3 inflammasome. Chadchan et al. also showed that n-butyrate inhibited HDACs activity which is required for endometriotic cell growth. Moreover, Samartzis et al. found that the expression of HDAC-1 was significantly elevated in patients with endometriosis, which inhibited histone acetylation (Samartzis et al., 2013). In summary, we believe that gut dysbiosis leads to the deficiency of n-butyrate, and causes an increased expression of HDAC-1 which plays a prominent role in epigenetic regulation in endometriosis.

### 4.2 Inflammatory

The gut microbiota can cause changes in the body’s metabolic pathways and immune responses, leading to local and even systemic immune inflammatory responses, which results in the body under the state of low inflammation for a long time. Endometriosis is a chronic inflammatory disorder that has been widely confirmed, and the role of the gut microbiome in inflammatory disease is now well characterized. Recently, a study showed that endometriotic lesions volume were significantly smaller and the inflammatory factors such as IL-1β, TNF-α, IL-6, and TGF-β1 in peritoneal fluid were obviously reduced in mice treated with broad-spectrum antibiotics or metronidazole than those in control mice. Metronidazole may inhibit endometriotic lesion growth in mice by reducing *Bacteroides* growth. They also found endometriotic lesion growth and inflammation were restored in mice gavaged with feces from mice with endometriosis in metronidazole-treated mice (Chadchan et al., 2019). Obviously, the research suggested that gut microbiota is related to endometriosis and promotes endometriotic lesion progression.

Shan et al. demonstrated that the abundance of *Streptococcus* in the stage III/IV endometriosis patients was higher than that in the healthy people, and the level of serum IL-8 was significantly elevated (Shan et al., 2021). In addition, previous studies have found that pro-inflammatory cytokines such as NF-κB, IL-1, IL-8 and cyclooxygenase-2 (COX-2) were inducibly overexpressed by *Streptococcus bovis* (Abdulamir et al., 2010). The overexpression of COX-2 increased the synthesis of prostaglandin E2 (PGE2), which is an important inflammatory factor inducing chronic pelvic pain of endometriosis. It can be seen that regulating the gut flora can be used as a target for the treatment of chronic pelvic pain in patients with endometriosis. Furthermore, studies have shown that inhibition of COX-2 could decrease survival, migration and invasion of endometriotic cells (Banu et al., 2008). Cao et al. found that the abundance of *Ruminococcaceae* was reduced in endometriotic rats compared to the control group (Cao et al., 2020). Studies have shown that there is a negative correlation between *Ruminococcaceae* and apoptosis of intestinal epithelial cells and IL-6 levels in mice (Meng et al., 2019). We confer that decreased abundance of *Ruminococcaceae* may exacerbate pelvic inflammation. Khan et al. reported that *Escherichia coli* exists in the menstruation of the patients of endometriosis, and refluxes to the pelvic cavity causing an increase in endotoxin in the peritoneal fluid, which activates TLR inflammatory signaling pathway to stimulate secretion of pro-inflammatory factors (Khan et al., 2010, 2012, 2014). The high levels of pro-inflammatory factors in the pelvic cavity, such as TNF-α, IL-6, IL-8, rapidly accumulate under the influence of activated peritoneal macrophages, resulting in an inflammatory environment of the pelvic cavity, which in turn stimulates the secretion of related adhesion molecules and promotes the implantation and adhesion of endometrial debris (Kim et al., 2012). Thus, microbial-induced inflammatory responses may promote ectopic adhesion, implantation, infiltration and growth of endometrial debris, and ultimately lead to the formation of endometriotic lesions.

### 4.3 Estrogen-gut microbiome axis in endometriosis

Although the pathological mechanism of endometriosis has not yet been elucidated, a large number of epidemiological and clinical studies have shown that endometriosis is an estrogen-dependent disease. Estrogen can promote cell adhesion, invasion, and proliferation of ectopic lesions, inhibit apoptosis, and maintain the inflammatory response (Ferrero et al., 2014; Mori et al., 2019). Estrogen can exert its biological function by binding to the estrogen receptor (ER) on the endometrium. ERs include classical nuclear receptors (ERα and ERβ), and non-classical membrane receptors (GPER1) (Borella et al., 2021). After ERs are activated, they can bind to the estrogen response element (ERE) which is the specific DNA sequence in the promoter region, or interact with other transcription factors in the nucleus to regulate the transcription of target genes. In addition, ERs regulate the genes targeted by estrogen lack ERE-like sequences such as NF-κB, activator protein 1 (AP-1) and stimulating protein-1 (SP-1) through protein–protein interactions with other transcription factors and response elements. In addition to the well-known genomic actions of estrogen, there are also non-genomic actions of estrogen. They can work through ERα, GPER1, mitogen activated protein kinases (MAPK), PI3 kinases (PI3K), cAMP and so on (Fuentes and Silveyra, 2019). However, the non-genomic actions of estrogen in endometriosis are still controversial, and further research is needed.

**Figure 3:**
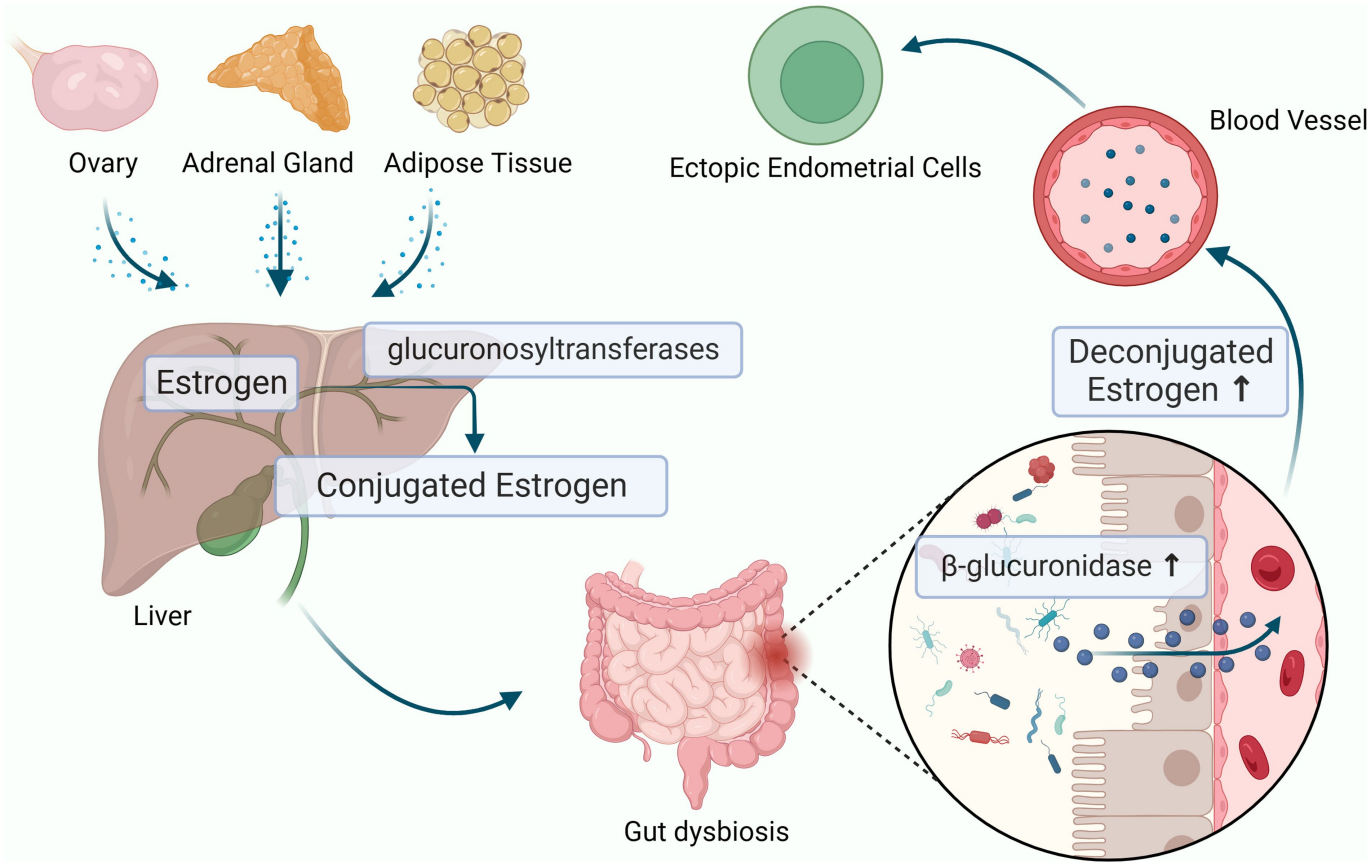
Estrogen-Gut microbiome axis in endometriosis. The dysbiosis of gut microbiota can produces beta-glucuronidase, which increase the level of circulating estrogen through the enterohepatic circulation and cause endometriosis (the figures in this review were created with BioRender.com).

At the same time, experimental studies have shown that the gut microbiota can affect estrogen metabolism and cause related diseases (Yang et al., 2017) (Figure 3). The estrogen in the human body mainly comes from the ovary, adrenal gland and adipose tissue, and then enters the blood circulation until it is metabolized in the liver. In the liver, estrogen combines with its metabolites resulting in conjugated estrogen, which is then metabolized into water-soluble molecules that are excreted in the urine and feces. Gut flora imbalance produces beta-glucuronidase, which prevents the combination of estrogen and glucuronic acid to reduce the inactivation of estrogen and to increase the level of circulating estrogen through the enterohepatic circulation thereby significantly stimulating the growth of ectopic lesions and participating in the pathological process of periodic bleeding of endometriosis (Plottel and Blaser, 2011; Flores et al., 2012; Shen et al., 2012; Baker et al., 2017; Wei et al., 2023). Previous studies have reported that *Clostridia* and *Ruminococcacaeae* spp. produce active estrogens through deconjugation, influencing estrogen-dependent diseases (Bhatt et al., 2017). Uccello et al. found that *Lactobacillus* and *Bifidobacterium* can reduce the proportion of β-glucuronidase-producing bacteria in the intestine, leading to a decrease in estrogen reabsorption rate and reducing the risk of estrogen metabolism diseases (Uccello et al., 2012). Shan J et al. found that the abundances of *Blautia* and *Dorea* were significantly increased which were positively correlated with estrogen level in patients with stage III/IV endometriosis (Shan et al., 2021). However, the mechanisms by which the gut microbiota increases the levels of estrogen promote endometriosis development remain to be further explored.

### 4.4 Gut-brain axis in endometriosis

The microbiome-gut-brain axis (MGB) has attracted increasing attention and refers to the bidirectional communication between gut microbes and the central nervous system. This axis is considered a key regulator of immune, neural, endocrine and metabolic signaling pathways that maintain homeostasis. MGB dysregulation has been shown to be involved in many diseases such as irritable bowel syndrome (IBS) (Mayer et al., 2022), depression (Averina et al., 2020), pain (Guo et al., 2019), and PCOS (Insenser et al., 2018; Zhang J. et al., 2019; Liang et al., 2021).

The microbiota can affect the production and secretion of neurotransmitters in various ways, including γ-Aminobutyric acid (GABA), norepinephrine, dopamine, and serotonin and so on (Strandwitz, 2018; Yang et al., 2021). These neurotransmitters can affect the hormone levels of the host. Therefore, changes in the composition of gut microbiota may be the reason for changes in host hormone levels. Gut bacteria are closely connected to the functioning of the hypothalamus-pituitary-adrenal (HPA) axis. Previous studies have shown that hypocortisolemia is a biomarker of dysfunction of the HPA axis in women with endometriosis (Petrelluzzi et al., 2008; Quinones et al., 2015). We speculate that the role of gut microbiota in endometriosis is related to the impact on the HPA axis. Recent study found that the increasing GABA-producing bacteria might increase the level of GABA in women with PCOS, and act on the receptors of GnRH neurons in hypothalamus to influence the secretion of LH and FSH (Liang et al., 2021). Salliss and colleagues suspected that because gut dysbiosis directly and indirectly stimulates GnRH neurons in the hypothalamus, resulting in hypothalamus-pituitary-ovarian (HPO) axis dysregulation and influence the secretion of sex hormones including GnRH, LH, FSH, and estrogen. This dysbiosis could ultimately impact estrogen-associated disorders, such as endometriosis (Salliss et al., 2021). However, the above research area of endometriosis has been less well explored, and is worth exploring in more detail.

Furthermore, over the past few decades, many researches have shown that emotional disorders are related to changes in the balance of neurotransmitters in the brain. For example, major depressive disorder (MDD) and anxiety are associated with imbalances in the serotonin system and abnormalities in the HPA axis (Frankiensztajn et al., 2020). These diseases are also highly correlated with microbial disorders. Chronic stress such as anxiety and depression are common in patients with endometriosis, with an average incidence of approximately 41.78% (Brasil et al., 2020). Xu et al. divided the endometriosis patients into chronic stress and control groups by generalized anxiety disorder-7 (GAD-7) and patient health questionnaire-9 (PHQ-9) questionnaires. They found that the level of *Paraprevotella*, *Odoribacter*, *Veillonella*, and *Ruminococcus* were significantly reduced in chronic stress group compared to the control group at the genus level. They also demonstrated that the dysbiosis of gut microbiota may influence the psychological state of patients with endometriosis through the inflammatory pathway of gut-brain axis (Xu et al., 2017). In addition, many researchers have shown that gut microbiota dysbiosis play an important role in depression by the decreasing of SCFAs (Deng et al., 2019). Silva et al. considered that the anti-inflammatory property of SCFAs was related to the development of depression (Silva et al., 2020). The level of SCFAs also reduce in patients with endometriosis. Given that SCFAs can regulate the central nervous system directly and indirectly, ultimately affecting patient behavior and cognitive function. A thorough understanding of how SCFAs participate in gut-brain interactions may help identify new targets for the treatment of psychological problems related to endometriosis.

**Figure 4:**
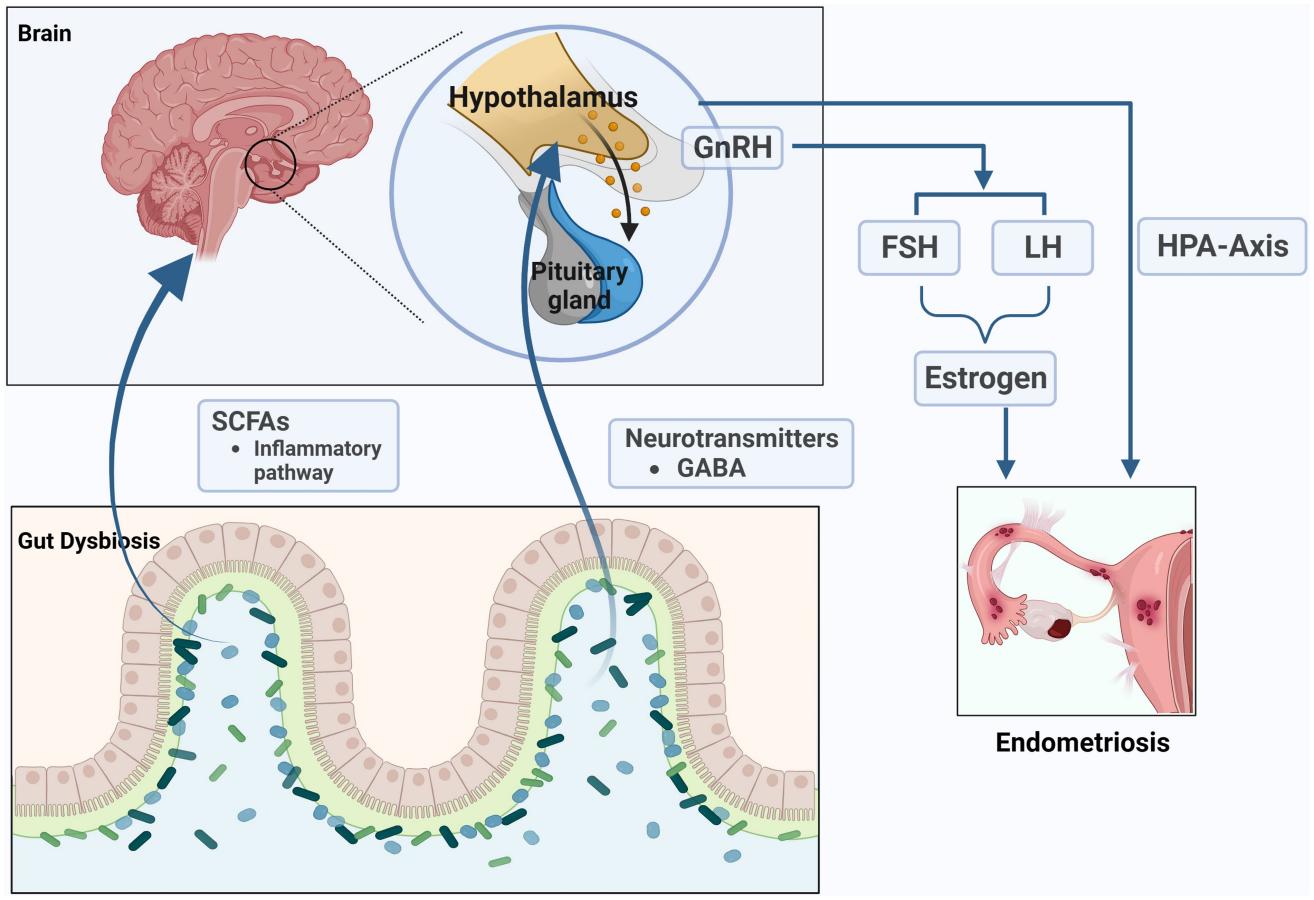
The microbiome-gut-brain axis (MGB) in endometriosis. The microbiota can affect the production and secretion of SCFAs and neurotransmitters such as γ- Aminobutyric acid (GABA), which can affect the hormone levels of the host. The role of gut microbiota in endometriosis is related to the impact on the hypothalamus–pituitary–adrenal (HPA) axis and hypothalamus-pituitary-ovarian (HPO) axis (the figures in this review were created with BioRender.com).

The proposal of the MGB axis may provide a new perspective for better understanding endometriosis and exploring its pathogenesis and treatment methods (Figure 4). However, further research is needed on the causal relationship between the MGB axis and the pathogenesis of endometriosis. In recent years, the importance of the MGB axis has been widely recognized, and its dysfunction can lead to various diseases (Zhu et al., 2022). Numerous studies are exploring the physiological role of the MGB axis, hoping to find intervention or treatment targets for endometriosis from this perspective.

## 5 Gut microbiota and the malignant transformation of endometriosis

In 1925, Sampson reported that malignant tumors and endometriosis coexist in the same lesion for the first time. Endometriosis will generate malignant transformation under certain conditions or incentives. The malignant transformation rate of endometriosis is about 0.5–1.0% (Kobayashi et al., 2007; Murakami et al., 2020), and it usually occurs in the ovaries. When ovarian cancer is associated with endometriosis, and the cancer tissue is adjacent to the ectopic lesion, and there are persistent changes from ectopic endometrium to atypical hyperplasia and malignancy, it is regarded as “endometriosis-related ovarian cancer (EAOC).” It is widely accepted that the precursor of ovarian clear cell carcinoma (CCC) and ovarian endometrioid carcinoma is endometriosis.

The pathogenesis of the EAOC remains unclear. Many scholars believe that the essence of tumor is an inflammatory process. It has been reported that chronic inflammatory diseases and infections are responsible for approximately 25% of cancers worldwide (Hussain and Harris, 2007). Several studies have suggested gut microbiota play an important role in the development of cancer (Zhou et al., 2019; Laborda-Illanes et al., 2020; Borella et al., 2021) and so on. Gut bacteria can upregulate the TLR, and activate NF-kB, which can lead to the release of IL-6, IL-12, IL-17, IL-18 as well as TNF-α, and finally play a critical role in triggering persistent inflammation in the tumor microenvironment (Rutkowski et al., 2015; Laborda-Illanes et al., 2020). Zhou et al. found that the diversity and richness indexes of the microbiota were significantly decreased in ovarian cancer tissues compared to tissues from normal distal fallopian tubes. They also found that inflammation-associated signaling pathways were dramatically activated in ovarian cancer tissues, such as NF-κB signaling pathway, Cytokine-cytokine receptor interaction, and Chemokine signaling pathway et al. (Zhou et al., 2019). Moreover, Wang et al. found that LPS were present in the cancerous ovarian tissue (Wang et al., 2020). LPS stimulates TLR4 to cause immediate interaction between PI3K and MyD88, resulting in the phosphorylation of AKT, and ultimately promoting cell proliferation, invasion, and metastasis (Park et al., 2017). Abnormal activation of PI3K/AKT/mTOR pathway has been widely reported in the malignant transformation of tumors including EAOC (Murakami et al., 2020). This pathway has been highlighted as a biomarker for CCC therapy (Yachida et al., 2021). It suggested that the microbiota may be involving the initiation and progression of ovarian cancer via influencing the local inflammation or stimulating PI3K/AKT/mTOR pathway.

In addition, high level of estrogen is one of the high-risk factors for the malignant transformation of endometriosis. Estrogen dysregulation promote ectopic endometrium hyperplasia and malignant transformation through the increase of aromatase expression and activity. As mentioned above, gut dysbiosis can increase the circulation levels of estrogen and promote inflammation, in turn, inflammatory responses, along with the known hormonal dysregulation in endometriotic implants, may drive carcinogenesis (Dawson et al., 2018). Finally, we consider that the gut microbiota is likely to be involved in the malignant transformation of endometriosis, but it is still unknown how gut microbiota influence malignant transformation of endometriosis. Many researches are needed to prove whether the process of the malignant transformation of endometriosis can be controlled by the regulation of the flora.

## 6 Gut microbiota and endometriosis-associated pain and infertility

Pain is the main symptom of endometriosis, mainly manifested as secondary dysmenorrhea, back pain, non-menstrual pelvic pain and CPP, which is present in 50–80% of patients with endometriosis (Taylor et al., 2021). The severity of pain is not necessarily proportional to the size of the lesion. Patients with severe adhesions of ectopic ovarian cysts may have no pain, while small scattered lesions in the pelvis can cause unbearable pain which usually seriously affects patients’ daily life, emotions, interpersonal relationships, and work. Reducing and managing pain is one of the fundamental goals of treating endometriosis. In recent years, there has been increasing interest in the study of endometriosis-associated pain, but its pathological mechanism has not been fully elucidated. The emerging roles of the gut microbiota in pain regulation have attracted increasing attention, which provides a new direction for the study of endometriosis-associated pain.

PAMPs are considered to be an important factor in peripheral sensitization under pain conditions. PAMPs are transferred into the blood circulation and bind to RPR expressed on immune cells and sensory neurons in DRG, and participate in peripheral pain sensitization (Guo et al., 2019). Studies have shown that the process of ectopic endometrial adhesions implanted in the peritoneum to form ectopic lesions will undergo neurovascular formation (Asante and Taylor, 2011). Increased density of small unmyelinated nerve fibers which were often used as nociceptors could be found in endometriotic lesions and were closely related to the generation of endometriosis-associated pain (Tokushige et al., 2006). Meanwhile, Liu et al. found that the density of transient receptor potential vanilloid 1 (TRPV1)-positive nerve fibers in ectopic endometrium was higher than that in control endometrium and was positively correlated with the severity of dysmenorrhea in women with endometriosis (Liu et al., 2012). TRPV1 is a non-selective cation channel that plays an important role in inflammatory hyperalgesia. The increased TRPV1-positive nerve fibers may integrate various stimuli on peripheral terminals or primary sensory neurons and generate hyperalgesia in endometriosis (Liu et al., 2012; Bohonyi et al., 2017). In addition, previous studies found LPS can bind to TLR4 to activate and sensitize nociceptive neurons in dorsal root ganglia (DRGs) which derived from gut microbiota, via a TRPV1-mediated mechanism (Diogenes et al., 2011). We can speculate that gut dysbiosis leads to massive influx of LPS into the blood circulation, activates TRPV1 and increases its expression, further drives peripheral nociceptor sensitization, and plays a role in endometriosis-associated pain. On the other hand, PAMPs can act on non-neuronal cells such as immune cells to release pro-inflammatory cytokines, including TNF-α, IL-1β, and IL-8, which are indirectly activated or sensitized primary sensory neurons in DRGs. It has been previously discussed that macrophages and nerve fibers are both detected in the endometriosis. TNF-α and IL-1β mainly secreted by macrophages are highly expressed in the peritoneal fluid of endometriosis patients which can lead to peripheral nerve hypersensitivity and hyperalgesia (Wu et al., 2017; Liang et al., 2018; Maddern et al., 2020). This suggest that gut dysbiosis could promote the release of pro-inflammatory cytokines which could induce peripheral sensitization and play an important role in endometriosis-associated pain.

There is a strong correlation between endometriosis and infertility. Between 25 and 50% of patients with endometriosis experience infertility, and up to 50% of infertile patients may suffer from endometriosis (Bjorkman and Taylor, 2019; Taylor et al., 2021). However, the specific interaction mechanisms of endometriosis and infertility are still far to be clearly elucidated. The gut microbiota may play an essential role in the pathogenesis of endometriosis-associated infertility, but is less well studied. The impact of the gut microbiota upon endometriosis-associated infertility is likely due to the effects of the sex hormones (He et al., 2021), unbalanced immune profile, pro-inflammatory status (De Rivero Vaccari, 2020), and endometrial receptivity (Benner et al., 2018; Wang et al., 2021). Recently, a two-sample Mendelian randomization (MR) study revealed that genetically proxied intestinal microbiota had potentially causal effects on infertility, including the genetically proxied *Family XIII AD3011 group*, *Ruminococcaceae NK4A214 group*, *Betaproteobacteria*, *Burkholderiales*, *Candidatus Soleaferrea*, and *Lentisphaerae* (Zhang et al., 2023). Given that endometriosis-related drug therapy renders useless for infertility treatment, the gut microbiota interventions and therapy may hold much promise in the treatment of endometriosis-associated infertility.

## 7 Diagnosis

Patients with endometriosis can present with longer incubation periods, diagnosing for a time delay of up to 4–11 years due to the atypical early symptoms and particularly limited but invasive diagnostic methods (Agarwal et al., 2019). The gold standard for the diagnosis of endometriosis is laparoscopic surgery with histologic examination after biopsy. However, the surgical diagnosis is unrealistic for all women suspected of endometriosis. It is an urgent need to find an effective minimally invasive or non-invasive candidate biomarker. With the increasing research on endometriosis and gut microbiota, the discovery of endometriosis-related candidate microorganisms has potential diagnostic value. Several gut microbial abnormalities have been identified in the women or murine models of endometriosis. Huang et al. revealed that the reduction of *Lachnospiraceae Ruminococcus* in the feces of patients might be a potential biomarker for endometriosis (Huang et al., 2021). One study found that two of fourteen patients with stage III/IV endometriosis had a gut microbiome dominated by *Escherichia*/*Shigella*, and further follow-up of these patients showed severe gut involvement and received segmental colectomy. However, the gut microbiota of patients in the control group did not show a similar composition. This infers that gut microbiome analysis may be a method for predicting bowel resection (Ata et al., 2019). A two-sample Mendelian randomization (MR) study confirms that a total of 8 gut microbiota taxa are associated with endometriosis, among which the Class-*Melainabacteria*, family-*Ruminococcaceae*, and genus-*Eubacteriumruminantium* had a protective effect on endometriosis, while order-*Bacillales*, family-*Prevotellaceae*, genus-*Anaerotruncu*s, genus-*Olsenella*, and genus-*RuminococcaceaeUCG002* may increase the risk of endometriosis. These taxa may be used for indirect diagnosis of endometriosis (Ji et al., 2023).

More and more evidence observed that gut microbiota may affect endometriosis through gut microbiota-derived metabolites, which provides a new direction for early diagnosis of Endometriosis. Chadchan et al. found Quinic acid was significantly upregulated in feces of mice with endometriosis, which can promote the cellular proliferation and endometriosis lesion growth (Chadchan et al., 2023). Ni et al. also screened four important differential metabolites, named chenodeoxycholic acid (CDCA), ursodeoxycholic acid (UDCA), ALA, and 12, 13 s-epoxy-9z, 11, 15z octadecatrienoic acid (12,13-EOTrE) in the fecal metabolomics of endometriosis mice, which can be used as potential biomarker for the non-invasive diagnosis and treatment of endometriosis in the near future (Ni et al., 2020). Further investigations are needed to identify the importance of the microbiome and metabolites in the diagnosis of endometriosis.

## 8 Treatment

Current treatment options for endometriosis include medical therapy of ovarian suppression and surgical resection of lesions. Nonsteroidal anti-inflammatory drugs (NSAIDs), oral contraceptive, progestins, GnRH analogs, aromatase inhibitor, and androgen analog have broad clinical application in the long-term management of endometriosis, but are not curative. Because of the high recurrence rate after surgical excision, the procedure is usually repeated many times, despite once during the entire endometriosis life should be stressed, ideally. Given the high recurrence rate after surgery and side-effects of drugs, further studies are needed to explore the following potential therapeutic approach in endometriosis.

### 8.1 Probiotics

Probiotics are a class of active microorganisms, which colonize the digestive system of the human and can promote the microecological balance of the host. Probiotics are mainly composed of *lactobacillus* and *Bifidobacterium*, which can enhance intestinal epithelial integrity, protect the intestinal barrier, modulate the immune system of the gastrointestinal mucosa and inhibit the growth of pathogenic bacteria (Kim et al., 2019). Probiotics have become a research hotspot and have beneficial effects on the treatment of many diseases (Yang et al., 2021).

Probiotic interventions that alter the type and abundance of gut microbiota, as well as human immune cells and immune molecules, have great potential as new targets for the treatment of endometriosis. Itoh Hiroyuki and colleagues found that *Lactobacillus gasseri* OLL2809 which is one of a probiotic *Lactobacillus* suppressed development of endometriosis via activation of NK cells in mice (Itoh et al., 2011). Additionally, a randomized, double-blind, placebo-controlled study showed that the tablet containing *L. gasseri* OLL 2809 could improve menstrual pain and dysmenorrhea and had no adverse effects in patients with endometriosis (Itoh et al., 2011). A pilot randomized triple-blind placebo-controlled trial found that oral lactobacillus could significantly alleviate endometriosis-associated pain (Khodaverdi et al., 2019). The aforementioned studies suggested that probiotics can not only inhibit the progression of endometriosis, but also are beneficial for endometriosis-associated pain relief without obviously side effects. Combined oral contraceptive with non-steroidal anti-inflammatory drugs (NSAIDs) (continuous dosing to prevent dysmenorrhea) or progestins with NSAIDs was the First-line therapy for endometriosis-associated pain, which are often accompanied by significant and unwelcome side effects (Taylor et al., 2021). The recurrence rate of endometriosis-associated pain is as high as 40–50% after surgery (Vercellini et al., 2009). Probiotics have great potential as new targets for the treatment of endometriosis-associated pain. However, research related to this filed is still in its initial exploratory stages, reminding us of the need for more preclinical and clinical work to investigate the role of probiotic therapy in endometriosis.

### 8.2 Diet

The therapeutic potential of dietary interventions and dietary modification for patients with endometriosis has received increasing attention as complementary therapy and self-management. Diet is a shared substrate for host and gut microbes, which can change the composition and metabolic activity of the gut flora. Omega-3 polyunsaturated fatty acids (Omega-3 PUFAs) are essential fatty acids that humans should obtain from their diets, mainly from fish, seaweed and nuts, and are involved in regulating intestinal immunity and maintaining intestinal homeostasis, including docosahexaenoic acid (DHA), eicosapentaenoic acid (EPA), alpha-linolenic acid (ALA), and docosapentaenoic acid (DPA). Omega-3 PUFAs can affect the gut microbial community. The previous study reported that omega-3 PUFAs could decrease LPS-producing bacteria (such as *E. coli*) while increasing LPS-suppressing bacteria (such as *Bifidobacterium*), leading to the reduction of inflammatory cytokines (TNF-α, IL-1β, and IL-6) (Kaliannan et al., 2015). Growing evidence suggests that omega-3 PUFAs have inhibitory effects on endometriosis. Animal studies suggested that omega-3 PUFAs suppressed the development of endometriosis via their anti-inflammatory effects (Tomio et al., 2013; Attaman et al., 2014). A prospective study among premenopausal women demonstrated that long-term intake of omega-3 PUFAs reduced the incidence of endometriosis (Missmer et al., 2010). ALA is an omega-3 PUFAs which is commonly found in vegetable oil seeds. The abundance of ALA was significantly decreased in the feces of endometriosis mice which were influenced by gut dysbacteriosis (Ni et al., 2020). The exogenous supplementation of ALA could obviously improve the abundance of *Lactobacillus*, *Bacteroides*, *Muribaculum*, *Clostridium_ sensu_ stricto_ 1*, and *Bifidobacterium*, and reduce the level of LPS in the abdominal cavity and the aggregation of peritoneal macrophages in endometriosis mice (Ni et al., 2021). In a rabbit model of endometriosis, ALA supplementation could reduce the concentrations of PGE2 and the diameter of endometriotic lesions (Covens et al., 1988). It is speculated that Omega-3 PUFAs modulation of the gut microbiota may contribute to the prevention and treatment of endometriosis.

In addition, a dietary therapy called low-FODMAP which is a diet low in fermentable oligo-, di-, monosaccharides, and polyols could alter the diversity of gut microbiota and their metabolites. A low-FODMAP diet could reduce fecal LPS by modulating gut microbial composition and improves intestinal barrier function (Zhou et al., 2018). Some studies have demonstrated the effectiveness of this dietary intervention in the treatment of irritable bowel syndrome (IBS) through potential gut microbiota-related pathways (Hustoft et al., 2017; Su et al., 2019; Milajerdi et al., 2020). Patients with endometriosis have a higher incidence of gastrointestinal symptoms, up to 90%, and often suffer from IBS before diagnosis of endometriosis (Seaman et al., 2008; Maroun et al., 2009). In a retrospective analysis of a cohort study, it is found that a low-FODMAP diet is beneficial in reducing bowel symptoms in women with endometriosis (Moore et al., 2017). This dietary intervention may be an emerging approach to relieve gastrointestinal symptoms of endometriosis. Another research showed that after a low-FODMAP diet for 3 weeks, symptoms consistently improved in patients with IBS, but fecal bacteria (Actinobacteria, Bifidobacterium, and *Faecalibacterium prausnitzii*), total SCFAs and n-butyric acid were significantly reduced which drastically affected gut health (Hustoft et al., 2017). To avoid these problems, it is recommended that patients selectively reintroduce eliminated food after treatment with a low-FODMAP diet (Mayer et al., 2022). However, a low-FODMAP diet in patients with endometriosis requires further research. Diet is one of the main factors affecting and regulating the composition of intestinal flora, thus, it is important to understand how the diet affects the intestinal flora. Although its exact mechanism remains unclear, diet intervention is a crucial supplemental treatment of endometriosis.

### 8.3 Fecal microbiota transplantation

FMT means transplanting the infusion of a fecal suspension from the health donor to the subject. FMT can directly improve the disorders of intestinal flora and develop a new treatment pathway to a series of microbial-mediated diseases, such as IBS (Johnsen et al., 2018), ulcerative colitis (Ding et al., 2019), depression (Doll et al., 2022), metabolic syndrome (Kootte et al., 2017). FMT has been widely recognized in the treatment of *Clostridium difficile* infection (CDI), and the effective rate of FMT for recurrent and refractory CDI is more than 90% with few adverse reactions (Quraishi et al., 2017). However, there is no experimental report of FMT in the treatment of endometriosis currently, which needs further exploration.

## 9 Limitations and future directions

Currently, much of the ongoing research is focused on the correlation between the gut microbiota and endometriosis, however, further systematic studies are needed to elucidate the underlying mechanisms. The rapid development of modern genome sequencing technology has transformed researchers’ understanding of microscopic organisms within a short period. However, due to the large number and complex composition of gut microbiota, researchers are unable to accurately grasp data on all microorganisms in the intestine, and may ignore undetected or low abundance bacterial species. Therefore, further research on the gut microbiota and microbiome-derived metabolites will not only deepen our understanding of endometriosis, but also provide a theoretical basis to become a new treatment method in this field. Although endometriosis is subdivided into three categories histologically, including superficial peritoneal endometriosis, deeply infiltrating endometriosis, and ovarian (cystic) endometriosis, endometriosis lesions do not exist in isolation leading to extensive lesions coexisting with heterogeneous clinical manifestations and ultimately leading to pain and infertility. Future work will need to elucidate further mechanisms by which specific microbiota compositions or microbiota-mediated metabolites regulate endometriosis, either with pelvic pain being the predominant symptom or with infertility being the dominating symptom, searching for earlier, safer, and more sensitive diagnostic methods and more effective treatment modalities. Besides, bioinformatics analysis, such as analysis frameworks for single-cell RNA sequencing (Hu et al., 2023; Meng et al., 2023), deep learning predictive model (Gao et al., 2023; Wang et al., 2023), constantly updated microbiome-gene database, drug metabolism and biotransformation of the gut microbiota, combined with particular gut microbiota compositions determined could be utilized for early screening, as well as risk stratification according to endometriosis severity, in order to timely interventions and precise treatment. Given current surgery treatment modality with high recurrence rate and medical treatment with severe side effects, novel interventions that target specific microorganisms after microbiologic identification could be developed to reduce the potential side effects of treatments and give an effective systemic therapy for endometriosis.

## Author contributions

CG: Writing – original draft. CZ: Supervision, Writing – review & editing, Conceptualization, Formal analysis, Validation, Visualization.
